# ASCEND-UNet: An Improved UNet Configuration Optimized for Rural Settlements Mapping

**DOI:** 10.3390/s24175453

**Published:** 2024-08-23

**Authors:** Xinyu Zheng, Shengwei Pu, Xingyu Xue

**Affiliations:** 1 College of Mathematics and Computer Science, Zhejiang A & F University, Hangzhou 311300, China; shengweipu@126.com (S.P.); xyxue@zju.edu.cn (X.X.); 2 Key Laboratory of State Forestry and Grassland Administration on Forestry Sensing Technology and Intelligent Equipment, Hangzhou 311300, China; 3 Key Laboratory of Forestry Intelligent Monitoring and Information Technology of Zhejiang Province, Hangzhou 311300, China

**Keywords:** remote sensing, UNet, dilated convolution, attention mechanism, rural settlements

## Abstract

Different types of rural settlement agglomerations have been formed and mixed in space during the rural revitalization strategy implementation in China. Discriminating them from remote sensing images is of great significance for rural land planning and living environment improvement. Currently, there is a lack of automatic methods for obtaining information on rural settlement differentiation. In this paper, an improved encoder–decoder network structure, ASCEND-UNet, was designed based on the original UNet. It was implemented to segment and classify dispersed and clustered rural settlement buildings from high-resolution satellite images. The ASCEND-UNet model incorporated three components: firstly, the atrous spatial pyramid pooling (ASPP) multi-scale feature fusion module was added into the encoder, then the spatial and channel squeeze and excitation (scSE) block was embedded at the skip connection; thirdly, the hybrid dilated convolution (HDC) block was utilized in the decoder. In our proposed framework, the ASPP and HDC were used as multiple dilated convolution blocks to expand the receptive field by introducing a series of dilated rate convolutions. The scSE is an attention mechanism block focusing on features both in the spatial and channel dimension. A series of model comparisons and accuracy assessments with the original UNet, PSPNet, DeepLabV3+, and SegNet verified the effectiveness of our proposed model. Compared with the original UNet model, ASCEND-UNet achieved improvements of 4.67%, 2.80%, 3.73%, and 6.28% in precision, recall, F1-score and MIoU, respectively. The contributions of HDC, ASPP, and scSE modules were discussed in ablation experiments. Our proposed model obtained more accurate and stable results by integrating multiple dilated convolution blocks with an attention mechanism. This novel model enriches the automatic methods for semantic segmentation of different rural settlements from remote sensing images.

## 1. Introduction

In recent years, China has implemented the rural revitalization strategy and focused on improving the rural living environment [1]. There is a upsurge in tearing down the old settlements and building new ones [2]. The architectural forms and spatial distribution of rural settlement agglomerations have been changed dramatically. The extraction of rural settlements information using remote sensing data is of great significance in guiding rural land planning and implementing rural revitalization strategies. In the rural areas of the Hangjiahu Plain, there are two types of rural settlements agglomerations: dispersed and clustered rural settlements. The dispersed rural settlements are old rural settlements built in previous decades. They have scattered spatial distribution and no obvious pattern [3]. On the other hand, the clustered rural settlements are modern villages, they are orderly arranged and have similar architectural forms with high building density [4]. Different rural settlement types are mixed and scattered in rural space.

There are challenges in distinguishing various types of rural settlements due to the absence of semantic segmentation networks and rural settlements datasets. Firstly, there is minimal discrepancy in the spectral, textural, and geometric characteristics of different rural settlements types from remote sensing images [4]. It would cause uncertainty to distinguish them without leveraging enough spatial information. Improved networks are needed for this task. Furthermore, deep learning networks can provide high-precision segmentation results for various semantic segmentation tasks [5,6], but these models were not designed to distinguish different rural settlements. Therefore, it is necessary to develop semantic segmentation models with rural settlement datasets for this task.

There are many studies on building extraction in urban areas. An urban building classification dataset contains individual building polygons derived from very high-resolution (VHR) satellite images [7]. Additionally, a detailed building model product was produced in China on a national scale through rooftop extraction and height estimation [8]. However, there are few studies that have focused on land-use classification and building extraction in rural areas [8]. For example, rural features have been extracted by multiresolution segmentation, with industrial land and rural settlements then distinguished by landscape metrics. This approach produced more accurate results than traditional object-oriented classification [4,9]. A SegNet semantic segmentation model was designed for rural construction land extraction with overall accuracy of 96% on Worldview-2 images [10]. There is a need to adapt various building extraction models from urban areas to rural areas.

At present, the fully convolutional network (FCN) including UNet [11], SegNet [12], and DeeplabV3 [13,14], enables end-to-end pixel-level classification of remotely sensed images. These models were widely used in extraction for buildings, roads, vegetation, etc. [15,16,17,18,19]. A UNet model was refined with an attention module for the building extraction task [20]. A UNet architecture with self-attention and reconstruction bias modules was proposed for the semantic segmentation of building rooftops [21]. A dilated residual convolutional network and a multi-scale context subnetwork were combined to create an end-to-end architecture for the extraction of rural settlements using Gaofen-2 images [22]. UNet and its improved versions provide alternatives with higher accuracy for the segmentation of rural settlements.

Characterizing spatial contextual features is essential for accurately extracting and distinguishing different rural settlement types, and the network improvement method with dilated convolution is a promising approach. There are multiple types of dilated convolution modules, such as atrous spatial pyramid pooling (ASPP) and hybrid dilated convolution (HDC). The ASPP maps different sizes of inputs to fixed lengths of outputs by pyramid pooling. ASPP consists of multiple convolutional kernels with different dilation rates that capture different ranges of contextual information, and the main function is to extract multi-scale features [23]. An ACU-Net based on ASPP and UNet was proposed for sea and land segmentation [24]. Previous studies concluded that the ASPP block could expand the receptive field, integrate multi-scale features in the network, and improve the recognition accuracy of feature boundaries, especially for multi-scale objects from complex backgrounds [25]. HDC is another form of dilated convolution module, which has been gradually applied to remote sensing image processing tasks. A built-up area extraction network based on a dual-attention transformer was proposed, and the HDC module was used to characterize the contextual information around the targets [26].

With wider receptive fields from multiple dilated convolutions, it is necessary to adjust the network to pay more attention to important areas and suppress information in irrelevant areas. The attention mechanism has been widely deployed in object extraction. The squeeze and excitation (SE) module was found to provide better segmentation of buildings of varying sizes and irregular shapes. Based on the SE module, there are three variants: the spatial squeeze and channel excitation block (cSE), channel squeeze and spatial excitation block (sSE), and the spatial and channel SE block (scSE). The scSE module is constructed of two submodules, the cSE and sSE [27]. Compared to the convolutional block attention module (CBAM), the scSE attention module is less computationally intensive. The ASPP module and SE module are added to the traditional UNet in the semantic segmentation task of remote sensing images [28].

It can be concluded that current research mainly utilizes the ASPP module, while there are fewer applications based on HDC. The UNet model embedded with multiple dilated convolution blocks is worth further study. The techniques mentioned above provide technical support for the extraction of different rural settlements. Moreover, the synergistic effect of the attention mechanism with multiple dilated convolutions is also worth investigating.

In this study, the main objective is a semantic segmentation task to distinguish two types of rural settlements with an improved UNet framework. The contributions of this work are summarized as follows:

(1) A novel ASCEND-UNet encoder–decoder architecture was designed. This model enabled end-to-end training and effectively extracted dispersed and clustered rural settlements in remote sensing images;

(2) The ASCEND-UNet model had three key aspects, the ASPP multiscale feature fusion module was added in the last encoder, the scSE block was embedded at the skip connection, and the HDC block was utilized in the decoder;

(3) The effectiveness of the proposed model was verified by a series of model comparisons and accuracy assessments with original UNet, PSPNet, DeepLabV3+, and SegNet. Moreover, the contribution of multiple dilated convolution blocks and attention mechanisms was discussed with the ablation experiments.

## 2. Methods

### 2.1. Study Area

**Figure 1:**
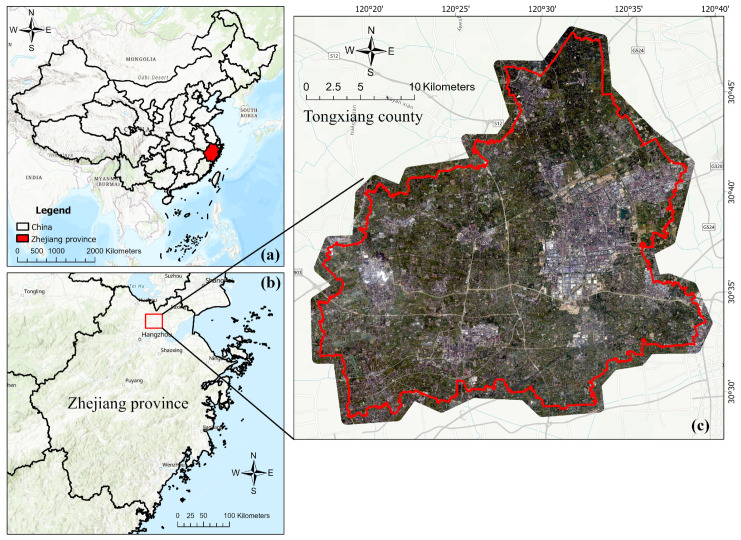
The study area of Tongxiang County and the Beijing-3 image of Tongxiang on 21 October 2023. (**a**) The map of China and Zhejiang province, highlighted in red, (**b**) the map of Zhejiang province, the study area is highlighted in red square, (**c**) the Beijing-3 image of Tongxiang County, the county boundaries are shown in red.

The study area is located in Tongxiang County, Zhejiang Province, China (Figure 1), with the geographic coordinates of 30°28′~30°47′ N latitude and 120°17′~120°39′ E longitude. Tongxiang County is in the center of the Hangjiahu Plain in the north Zhejiang Province, with a total area of 727.49 square kilometers. It has typical subtropical monsoon climate, with an average annual temperature of 16.5 °C and average rainfall of 1193 mm. The terrain is all flat, without hills. The land is fertile, which is suitable for the cultivation of rice and other crops.

Tongxiang County had been carrying out comprehensive rural land consolidation projects since 2009. By 2018, Tongxiang County had cumulatively approved 156 projects, relocated more than 20,000 rural settlements, and reclaimed 1390 hectares of construction land, of which 814 hectares were used for new rural settlement construction [3]. The dispersed rural settlements (DRSs) were demolished, and the newly built clustered rural settlements (CRSs) were formed. This was conducive to improving the rural living environment and increasing the area of arable land. Farmland that was once scattered has been concentrated and contiguous. As a result, the old-fashioned DRSs are mixed with uniformly planned new-fashioned CRSs. Therefore, it is necessary to distinguish these two types of rural settlements, and Tongxiang County is an ideal region to distinguish DRSs and CRSs with our proposed method.

**Figure 2:**
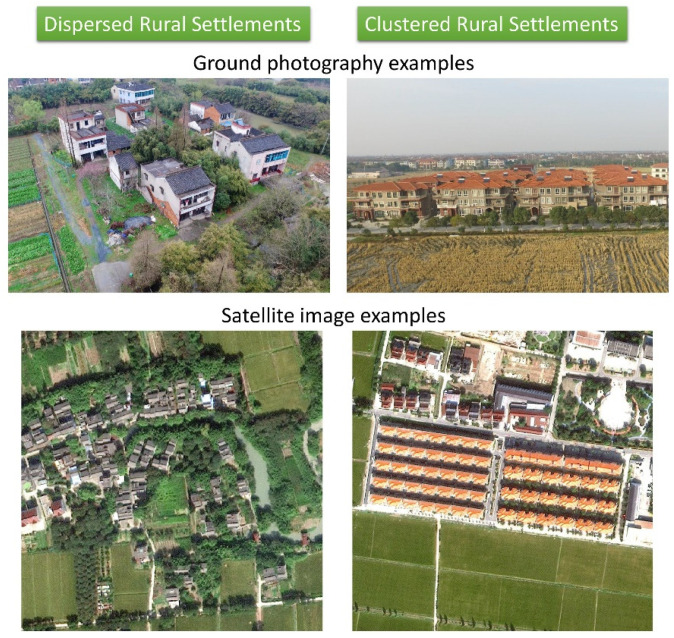
Image examples for dispersed rural settlements and clustered rural settlements.

The differences between a DRS and a CRS are not only in the architectural age but also in the spatial forms of rural communities (Figure 2). Their differences are exhibited by three aspects: composition, morphology, and location (Table 1).

The dispersed rural settlements (DRSs) are old-fashioned rural settlements that are scattered disorderly in their spatial distribution. As a result, the building density is low. DRSs are usually obscured by the surrounding vegetation (small vegetable fields, orchards, woodlands, etc.) The DRSs are mainly located alongside rivers and streams to support a small peasant economy.

In contrast, clustered rural settlements (CRSs) are standardized communities with an accurate and homogeneous architectural design, which results in efficient land-use management. CRSs have neatly arranged standardized settlement houses surrounded by less vegetation cover, and they are usually close to main roads for convenient transportation. Therefore, these two types of settlements have entirely different landscape characteristics [4,17].

### 2.2. Data and Preprocessing

The VHR satellite images used in our study were captured by the commercial satellite Beijing-3A, which were launched in 2021. The Beijing-3 images included four multispectral bands with a spatial resolution of 2 m and a panchromatic band with a sub-metric resolution of 0.5 m. After radiation correction and geometric correction, the multispectral bands and panchromatic band were fused to the VHR images with 0.5 m spatial resolution by the NNDiffuse pan-sharpening method. Image mosaic and clipping were operated on all VHR images containing red–green–blue (RGB) bands.

Ancillary data were collected in the form of land-use planning maps for rural villages (provided by the Bureau of Land and Resources, Tongxiang, China) and used as reference data in the image labeling step. All remote sensing images and vector data were adopted by the China Geodetic Coordinate System 2000 (CGCS2000) for projection.

### 2.3. Model Design for ASCEND-UNet

**Figure 3:**
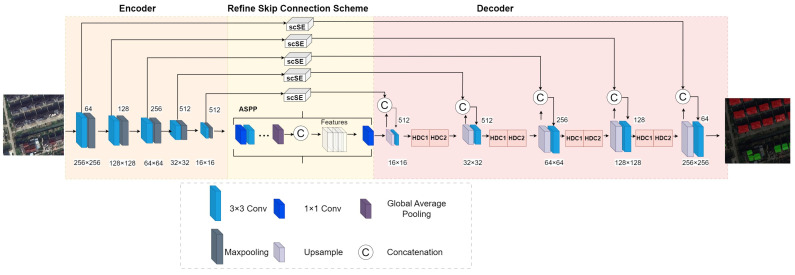
The structure of ASCEND-UNet network.

Our study improved the original UNet network structure by adding HDC, scSE, and ASPP blocks. The four convolutional layers in the decoder were replaced by HDC blocks with a dilated rate of (1, 2, 5), and HDC utilized the multi-scale information more efficiently when extracting the features, enabling the model to discriminate different settlement categories. The scSE block was used in the UNet skip connection layers to operate the attention mechanism on both the channel dimension and the spatial dimension. VGG16 was selected as the encoder in our network. In the last encoder, the ASPP multi-scale feature fusion block was embedded to capture the contextual information by dilated convolution at different dilated rates. ASPP increased the receptive field to process the feature information at different scales and improve the model’s ability to understand the contextual information. This ASCEND-UNet network structure could improve the semantic segmentation performance in rural areas and realize more accurate settlement segmentation. The ASCEND-UNet network structure is shown in Figure 3.

The specific structural parameters of ASCEND-UNet are outlined in Table 2. “Conv” stands for convolution, “Maxpool” for maximum pooling, “Upsample” for up-sampling, and “Stride” indicates the stride. This table lists the name of each layer in the network structure with the size of the output feature map.

### 2.4. HDC Block

The principle of HDC is to use a set of dilated rate convolutions in a continuous convolution layer instead of using convolutions with a fixed dilated rate. The HDC block expands the receptive field of the network and captures multiscale contextual information in a wider spatial scale, while also focusing on local detail information. The HDC fuses multiscale semantic information through an integration strategy [29].

**Figure 4:**
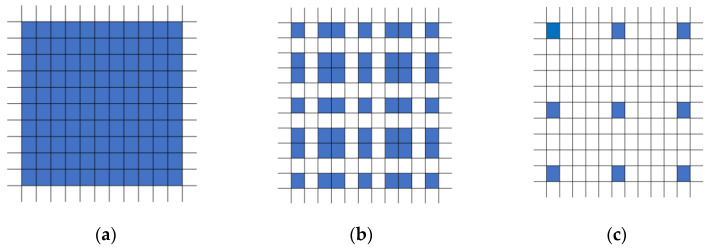
The HDC blocks with (**a**) dilate rate = 1, (**b**) dilate rate = 2, (**c**) dilate rate = 5.

HDC consists of three main features:

(1) Ensure that there is no common divisor greater than 1 between the dilated rates of the convolutions. Such settings can effectively cover each part of the input feature and avoid losing information due to dilated convolution;

(2) Set the dilated rate into a serrated structure, for example (1, 2, 5), the convolution kernel size is 3 × 3, as shown in Figure 4;

(3) The dilated rate needs to satisfy the formula:

(1) $$M_{i}=\max\;[M_{i+1}-2r_{i},M_{i+1}-2\left(M_{i+1}-r_{i}\right),r_{i}]$$

The $r_{i}$ means the dilated rate of layer i, and $M_{i}$ means the maximum dilated rate of layer i. Assume that there are n layers in total, then $M_{n}=r_{n}.$

The receptive field can be calculated as

(2) $$RF=\left(W-1\right)*\left(1-\frac{1}{r}\right)*{\left(\frac{K-1}{K}\right)}^{L-1}+1$$

where RF means the size of the receptive field, W is the width of the input feature map, r is the dilated rate, K is the width of the convolutional kernel, and L is the depth of the network.

**Figure 5:**
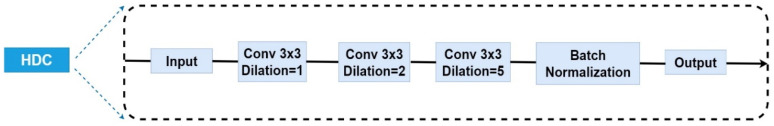
The structure of HDC block.

Through comparative experiments, we found that the best segmentation results can be achieved when the dilated rate is (1, 2, 5). The convolutional layers with dilated rates of (1, 2, 5) were connected sequentially, then the batch normalization layer and the ReLU activation function were used. The HDC block is shown in Figure 5.

### 2.5. scSE Block

The ASCEND-UNet proposed in this paper utilized the scSE block to address the problem of traditional UNet networks that cannot pay sufficient attention to the features of key regions in the image. In our rural settlement extraction scenario, spatial morphological features would be ignored by a traditional UNet, resulting in the possibility of inaccurate recognition of rural settlements.

**Figure 6:**
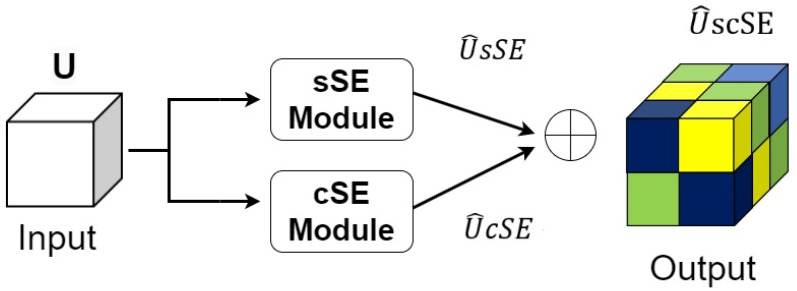
The structure of scSE block, the different colors of blocks represent the different weights.

The scSE attention mechanism block has a dual process on both the space and channel at the same time, consisting of the sSE module and the cSE module in parallel. The sSE module focuses on the spatial dimension, while the cSE module focuses on the channel dimension [27]. The scSE block enhances the representation of the features from both spatial and channel perspectives of rural settlements. The scSE block structure is shown in Figure 6.

The scSE block structure (Figure 6) started with an input feature map U (with H × W × C dimensions), then it entered the branching structure to extract both the spatial and channel feature information, after which these two features were summed up in Equation (3):

(3) $$\hat{U}scSE=\hat{U}cSE+\hat{U}sSE$$

**Figure 7:**
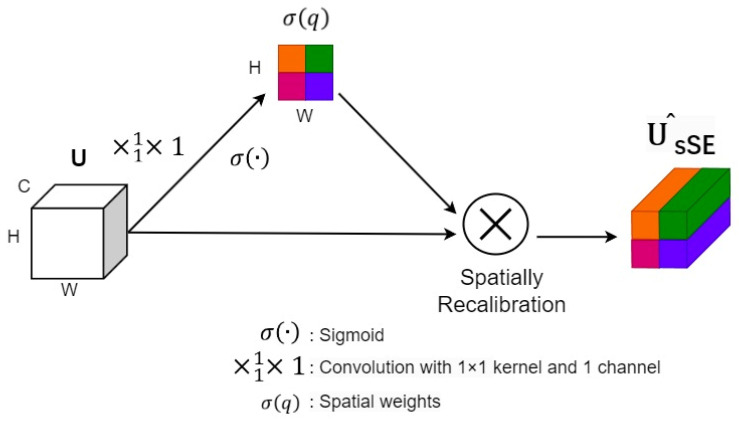
The structure of sSE block, the different colors of blocks represent the weights in different spatial locations.

In the sSE module (Figure 7), convolution with 1×1 kernel and 1 channel was performed to obtain an attention matrix of dimension (1, H, W). The sigmoid function was then used to generate the final weight matrix. Lastly, the weight matrix was multiplied with the original map in the spatial dimension.

**Figure 8:**
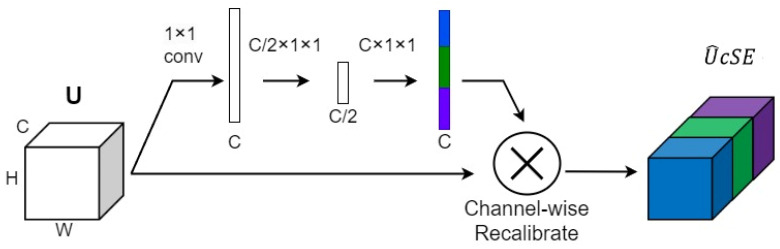
The structure of cSE block, the different colors represent different channel weights.

In the cSE module (Figure 8), first a 1×1 convolution was performed on the input feature map, then the number of channels of the input feature map was down-sampled from 1×1×C to 1×1×12C by a fully connected layer and ReLU activation function. Followed by normalization through another fully connected layer and a Sigmoid function, the dimension was up-sampled back to 1 × 1 × C. Finally, the weight map was multiplied by the original feature map in the spatial dimension to derive the spatial weights of different spatial locations.

### 2.6. ASPP Block

**Figure 9:**
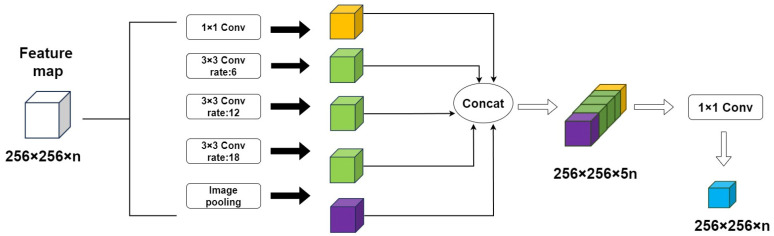
The structure of the ASPP block. The data dimension input the ASPP module is 256 × 256 × n, where n is the number of channels for input data at the current layer. In this study, n is 512 and 256 × 256 is the spatial dimension after resizing. The number of feature map dimensions for each branch output is 256 × 256 × n. Thus, the five branches yield a total of five feature maps in 256 × 256 × n dimensions. Finally, the five feature maps are superposed into a feature map with 256 × 256 × 5n dimensions. The different colors of blocks represent the output feature maps from different dilated convolution.

The ASPP multi-scale feature fusion module was embedded at the end of the ASCEND-UNet encoder, as the spatial morphology was different for the two settlement types in the remote sensing imagery. The ASPP block resampled the feature map with multiple dilated convolutions in parallel with different dilated rates. These resampled feature maps were integrated in parallel for the final output [30]. The ASPP block is composed of one 1 × 1 convolution layer, three dilated convolution layers, and one pooling layer, with a total of 5 branches (Figure 9). The first step is a 1 × 1 convolution layer to adjust the number of channels of the global averaged pooled feature maps, so that they have the same number of channels as the other feature maps generated by the ASPP block.

The second step employs three 3 × 3 convolution kernels with different dilated rates (6, 12, and 18, respectively).

The third step is down-sampling via adaptive global average pooling to compress the input feature map to a fixed-size feature map. This map is then up-sampled using a bilinear interpolation method, so the feature map is up-sampled to the same size as the other feature maps for feature fusion.

In the fourth step, all five feature maps from 5 branches are joined in the channel dimension to form a comprehensive feature map. To ensure the same weight of the feature maps from different dilated convolution outputs, the number of channels of each branch must be the same. In ASPP, the five feature maps are performed in parallel, unlike the convolutional serial operation of HDC.

### 2.7. Model Training and Prediction

**Figure 10:**
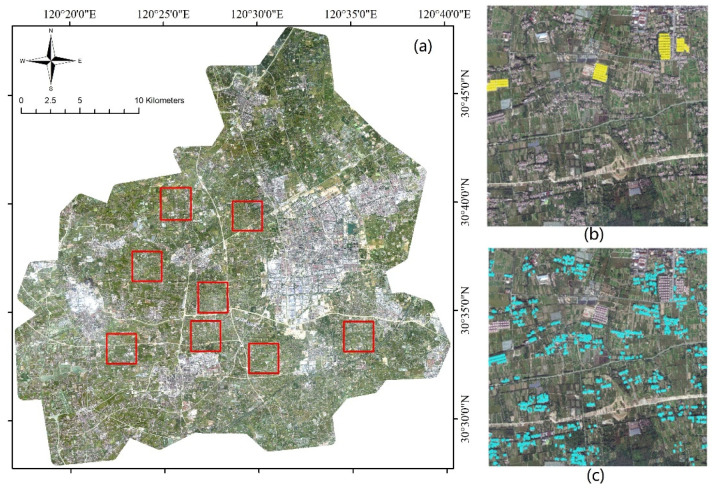
Eight square sample areas representing typical rural areas were randomly selected, and these sample areas avoided urban regions. (**a**) Square samples (red squares) of study area, (**b**) sample of CRS labels, (**c**) sample of DRS labels. The CRSs are represented in yellow and the DRSs are represented in blue.

After preprocessing, we randomly selected eight square sample areas to represent typical rural areas (Figure 10), each of which measures 5000 × 5000 pixels. Then the image dataset was input to the model for training. We cropped the sample area image into several patch datasets of the size 256 × 256.

The data enhancement strategy was used in this study [31]. Transformation operations such as random rotation, random cropping, and random inversion were performed on the images before model training, and multiple similar images were generated to increase the size of the dataset. The model mitigated overfitting issues by leveraging an enhanced dataset, thereby endowing the trained model with improved generalization capabilities. All images were randomly divided into training, test, and validation sets in the ratio of 8:1:1. Finally, 4866 training images, 608 test images, and 608 validation images were obtained. Validation images were used to adjust the model’s hyperparameters every 5 epochs.

In the labeling step, three categories were divided manually: DRS, CRS, and background. The background contained all land-use types other than rural settlements, including water bodies, farmland, forests, bare soil, roads, etc. The sample area and label samples are shown in Figure 10.

In our study, the original image was segmented into semantic categories of CRS, DRS, and backgrounds. The cross-entropy loss (CE loss) function was suitable to this task. CE loss effectively guides the model to distinguish settlement objects from the background by measuring the difference between the probability distribution predicted by the model and the probability distribution of the actual labels. The formula can be expressed as

(4) $$L=-\frac{1}{N}\sum_{i=1}^{N}\left[y_{i}\cdot \log\left(p_{i}\right)+\left(1-y_{i}\right)\cdot \log\left(1-p_{i}\right)\right]$$

where N is the total number of samples and $y_{i}$ is the true label of the *i* sample, which usually takes the value of 0 or 1. $p_{i}$ is the probability that the model predicts the *i* sample to be a positive class (value 1), the log is the natural logarithm.

### 2.8. Accuracy Assessment

The commonly used precision, recall, F1-score, and mean intersection over union (MIoU) were employed to evaluate the precision of rural settlement extraction results. True positives (TPs), false positives (FPs), and false negatives (FNs) indicate the number of correct, the number of incorrect, and the number of omissions, respectively, and k represents the number of categories. The specific formulas for the precision and recall are

(5) $$Precision=\frac{TP}{TP+FP}$$

(6) $$Recall=\frac{TP}{TP+FN}$$

The F1-score is a representation of the harmonic mean of precision and recall by the following formula:

(7) $$F1=\frac{2\times TP}{2\times TP+FN+FP}$$

MIoU is a model segmentation performance indicator; the larger the value represented, the better the segmentation results. The formula of MIoU is:

(8) $$MIoU=\frac{1}{k+1}\sum_{i=0}^{k}\frac{TP}{FN+FP+TP}$$

## 3. Results

### 3.1. Results and Comparison

**Figure 11:**
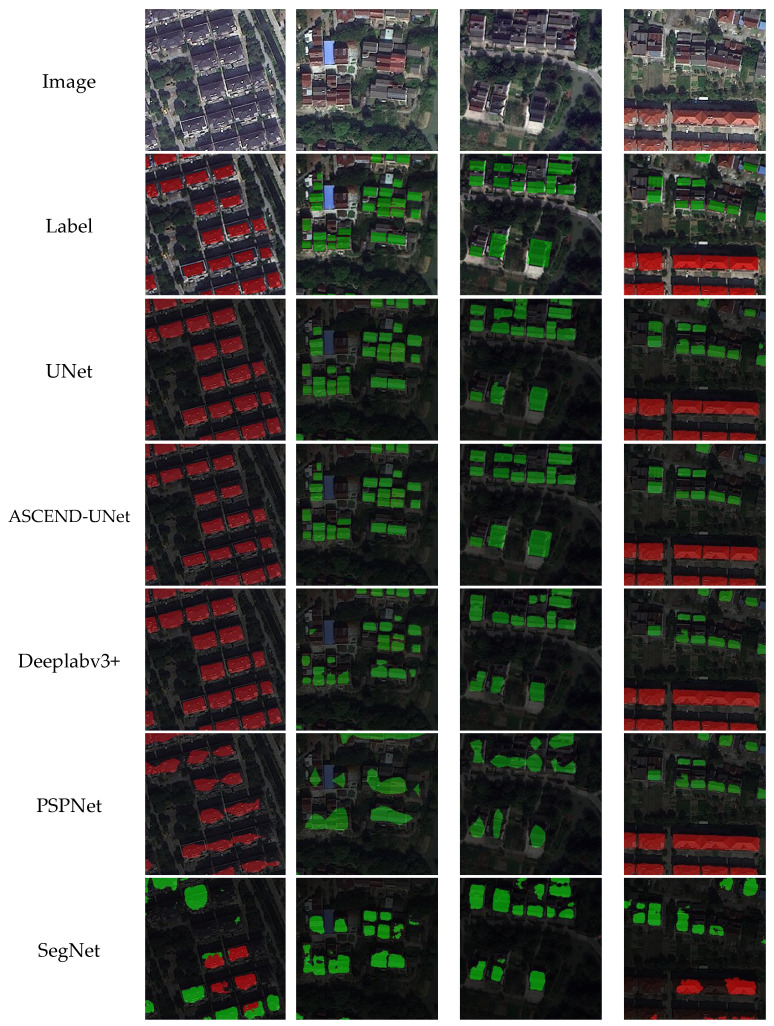
Results from different deep learning algorithms. The CRSs are represented in red and the DRSs are represented in green.

In this study, widely used semantic segmentation models were selected for comparison, including PSPNet, UNet, DeepLabV3+, and SegNet. The same training and test datasets were used to evaluate the models’ performance. The segmentation results obtained from different models are illustrated in Figure 11, where four sets of rural settlements with different shapes, sizes, and distribution patterns from remote sensing images are presented.

The visual comparison revealed that all the deep learning models had obtained building extraction results. However, there was a significant difference in the results from different models for different types of rural settlements and building edge depictions. For example, SegNet exhibited a high rate of misclassification when dealing with CRSs, as well as blurring of the building’s edges. PSPNet had omissions and significant deficiencies in the depiction of building edges. Deeplabv3+ worked better on CRSs but had omissions and edge blurring on DRSs with smaller building targets. The UNet model presented the best result among the original base models. However, there were still some details that needed to be improved. For example, there were a small number of omissions, and the accuracy of the edges could be improved in small, irregular building targets. Meanwhile, the proposed ASCEND-UNet obtained the best extraction accuracy and delineated the building boundaries more completely.

We also observed uncertainty in all algorithms. Due to the influence of the shadow of buildings and vegetation, this could lead to the inaccuracy of edge detection, especially in the area where the edges of the buildings were visually similar to the surrounding background.

**Figure 12:**
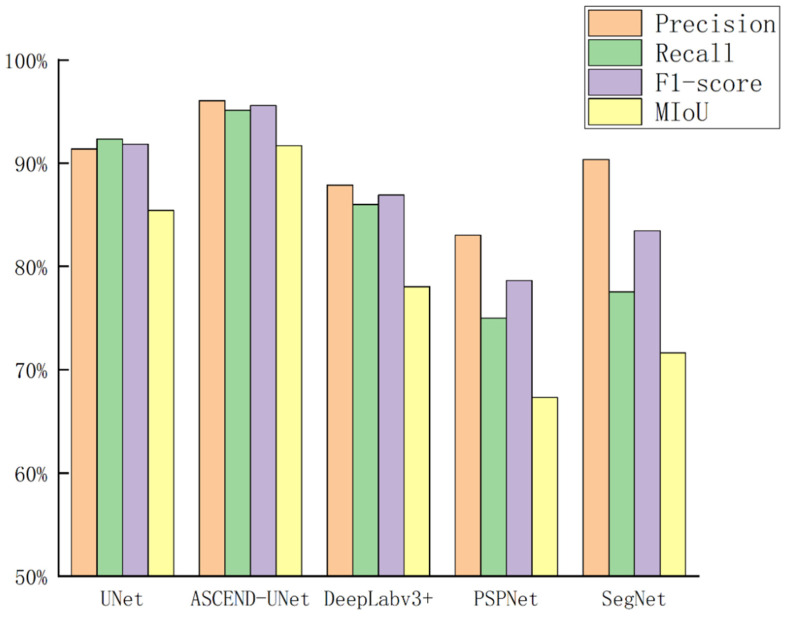
Accuracy assessment of different models.

By comparing the precision, recall, F1-score, and MIoU of each model in Table 3 and Figure 12, it was found that the ASCEND-UNet outperformed all other original models. The ASCEND-UNet achieved a significant improvement compared to the original UNet model, with 4.67%, 2.80%, 3.73%, and 6.28% increases in precision, recall, F1-score, and MIoU, respectively. The ASCEND-UNet captured the multi-scale spatial characteristics of rural settlements more effectively and provided more accurate results when distinguishing different types of them. In addition, ASCEND-UNet showed good robustness in complex background scenarios.

Table 4 presents the computing time of the proposed network and other methods. Our experiment was conducted on a 12 GB NVIDIA GeForce RTX 3060 GPU, and the Deeplabv3+ had the shortest time consumption. The UNet and our proposed ASCEND-UNet required a longer time for training and inference because they possessed more parameters and more complex model architecture. However, they exhibited superior performance in accuracy and stability.

### 3.2. Ablation Experiments

**Figure 13:**
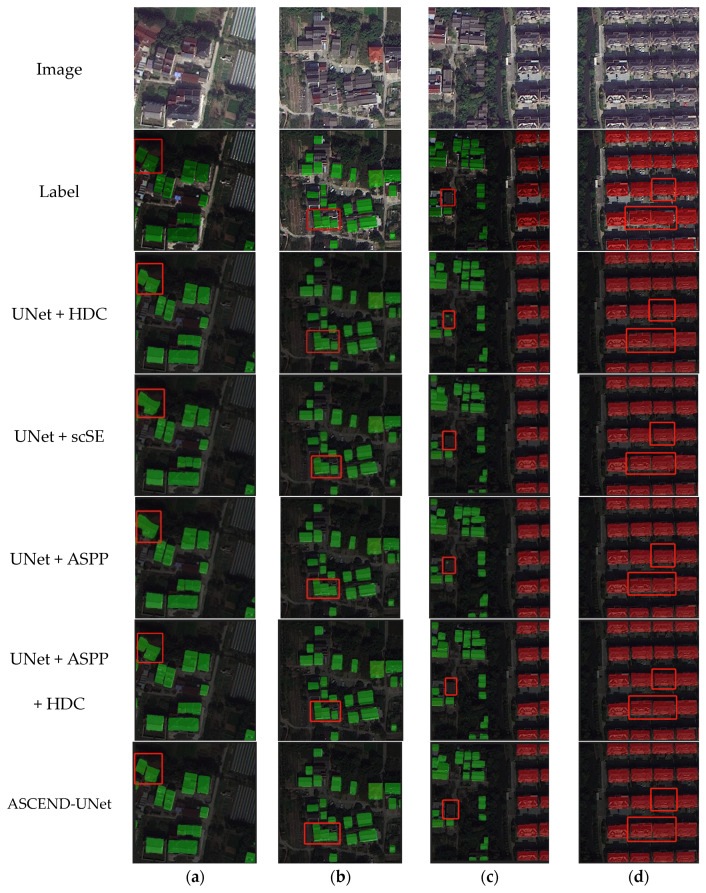
Segmentation result of ablation experiments, The CRSs are represented in red and the DRSs are represented in green. Both (**a**) and (**b**) represent the examples of DRSs, (**c**) represents the environment where CRSs and DRSs are mixed, and (**d**) represents the example of CRSs.

To evaluate the performance of the individual blocks in the ASCEND-UNet, we conducted a study that gradually incorporated the HDC, scSE, and ASPP blocks into the UNet network architecture. In Figure 13a,b, it was observed that the combination of UNet and HDC performed better in segmenting the building edges. In contrast, ASPP and scSE were found to be less effective in delineating building edges. While ASCEND-UNet showed the clearest and most accurate building edges. In Figure 13c, misclassification occurred when only the HDC and ASPP blocks were added. However, when HDC and ASPP were combined, some of the misclassified small objects were corrected. In Figure 13d, where the buildings are regular in shape and densely arranged, all the blocks incorporated into the UNet model depicted the edges of the buildings accurately, and there was no misclassification.

Our results showed that although the ASPP block has some advantages in feature fusion, it needed to be combined with other blocks for better edge delineating. The HDC and scSE blocks significantly improved the segmentation accuracy for complex scenes, especially in the building edge delineation.

The HDC, ASPP, and scSE block all had a positive impact on the building segmentation task (Table 5). With the ASPP and HDC blocks added, all the accuracy evaluation indicators showed significant growth. The HDC block contributed most significantly compared to the original UNet model; the precision increased by 4.60%, recall increased by 2.51%, the F1-score increased by 3.55%, and MIoU increased by 5.96%. It is worth noting that the improvement in recall was also significant when the ASPP and HDC blocks were added, with an increase of 0.33% compared to the ASPP block when used alone. This result suggested that the HDC block played an important role in enhancing the network’s ability to buildings segmentation.

## 4. Discussions

### 4.1. Distinguishing Different Types of Rural Settlements

In this study, the mixed problem of dispersed and clustered rural settlements was focused on to reflect the current situation of rural areas in the Hangjiahu Plain. The DRSs are old fashioned with a disorderly distribution. The DRSs also have poor rural housing and living conditions as a consequence of being built in the past. Self-sufficient small-scale peasant production requires a certain farming radius. As a result, the distribution of rural settlements is scattered, the settlement building plots are irregular, and the living areas are mixed with public land. The CRSs are new standardized rural communities constructed in recent years. The CRSs have orderly arranged houses, typically with one dwelling per household, without agricultural ancillary facilities around. There is planned public land for cultural and sports facilities, such as a conference hall. The CRSs are a recent outcome of rural land-use management and planning. CRSs integrate and consolidate several natural villages into a larger village community [4,22].

The evolution of different rural settlements not only represents changes over time but also signifies the transformation of rural production and living conditions [2]. The traditional small peasant economy has been replaced by large-scale intensive agricultural production, which extensively utilizes agricultural machinery. Rural life also has been changed, shifting its focus from being solely on agricultural production to social interaction, cultural activities, and sports as part of life’s requirements. Therefore, it is necessary to monitor these changes. The ASCEND-UNet model in our study provides a feasible semantic segmentation method for this task.

Distinguishing various types of rural settlement agglomerations is crucial for rural land planning, living environment improvement, and rural sustainable development [32,33]. For example, by mapping different types of rural settlements and conducting a statistical analysis of the proportion of DRSs and CRSs, rural management councils can make a plan to prioritise areas for rural living environment improvement. Moreover, the local governments can assess the rural living environment, monitor the progress of new rural construction, and evaluate farmland connectivity by analyzing the spatial distribution of different rural settlements. Given the various forms of rural settlements in the Shandong and Jiangsu provinces, and although this paper only considers two types, our proposed model can be adapted to other regions by creating a more diverse sample of datasets for rural settlements.

In a previous paper that took different types of rural settlements as research objects, an improved ResNet with dilated convolutions was proposed to obtain semantic segmentation results [22]. Although the previous paper’s method was different from ours, and the means of accuracy assessment were also different, we both obtained an accuracy higher than 90% (the previous research used area accuracy, while our research used pixels as the accuracy assessment unit). There is still a lack of alternative models to distinguish different types of rural settlements with suitable datasets. In our research, a novel ASCEND-UNet was designed for extracting different types of rural settlements. In contrast to the improved ResNet in the previous study, the ASCEND-UNet was based on the UNet framework which was easier to modify, and a modular improvement strategy was employed. Further research is still necessary to develop alternative methods for rural land feature extraction.

### 4.2. UNet Framework and Improvement

Unlike the deep learning models for building segmentation in previous studies, the goal of our study was to discriminate rural settlement agglomerations with different spatial distribution patterns, which not only requires the identification of buildings but also the recognition of spatial distribution patterns [34]. Compared to building segmentation, this goal is more difficult as the network is required to extract spatial contextual features. Although a large number of models and their improved versions have been proposed to improve segmentation accuracy in complex backgrounds, there is still a lack of models available to distinguish different types of rural settlements. Therefore, it is necessary to establish semantic segmentation models with rural settlement datasets for this task.

The UNet framework was initially used for medical image segmentation tasks [11]. A series of improvements based on the UNet network were proposed. For example, UNet++ introduced a dense skip-connecting structure to achieve better context awareness [35]. In our study, UNet achieved high accuracy as a baseline (Figure 11), especially in the CRS scenarios. Compared to PSPNet, DeepLabV3+, and SegNet, UNet had higher accuracy in settlement identification and edge depiction, which was in line with the findings of previous studies [34,36].

The UNet framework uses a symmetric “U-shape” structure with the same numbers for down-sampling (encoder) and up-sampling (decoder), and both sides of the network are symmetric to ensure the resolution of the input and output images remains constant. Another main feature of the UNet network is skip connections, which connect symmetric down-sampling and up-sampling levels. Feature information at different levels is then fused to reduce the loss of features. This method improves the segmentation results in the details. Therefore, in UNet framework applications, modular improvements can be made in the down-sampling and up-sampling processes, and the skip connection process. In our study, the encoder was enhanced with the ASPP block and the scSE block was embedded in the skip connection. Additionally, the HDC block was implemented within the decoder to facilitate more effective feature fusion.

### 4.3. Multiple Dilated Convolution Block with Attention Mechanism

The ASCEND-UNet proposed in our study recognized the rural settlements while classifying them into different spatial distribution patterns, which required the model to have spatial contextual feature extraction capabilities. We considered this requirement by including multiple dilated convolution blocks and attention mechanisms into the UNet framework.

Dilated convolution (e.g., HDC) introduces dilated rates, allowing the convolution kernel to skip pixels within the receptive field, thereby increasing the receptive field to capture a wider range of spatial context information. The size of the dilated rates determines the gap size of the convolution kernel to skip on the input feature map. A larger dilated rate leads to a larger receptive field but may also result in a reduction in resolution. However, the gridding problem arises when using dilated convolutions with the same dilated rate. This causes discontinuity of the information and the loss of small targets like rural settlements. To address this problem, the HDC has been proposed. HDC can use a series of arbitrary dilation rates, meanwhile expanding the receptive field of the network without adding additional modules [37]. This is important to the differentiation of different types of rural settlements. Moreover, it is important to note that the series of dilated rates should not have a common factor greater than 2 (e.g., 2, 4, 8, etc.), or else it will still lead to the gridding problem. In our study, HDC with a dilated rate of (1, 2, 5) was used in the decoder process.

The ASPP block also consists of dilated convolution to expand the receptive field but differs from the HDC block in the following ways. First, the ASPP structure is different from the HDC. The dilated convolutions of ASPP are constructed in parallel, while in HDC the dilated convolutions are constructed in a series [38]. Furthermore, in ASPP, we use larger dilated rates with common factors (6, 12, 18), so the ASPP block can capture wider range of spatial contextual information than HDC.

With wider receptive fields from multiple dilated convolutions, we adjusted the network with the scSE attention mechanism to pay more attention to important areas and suppress information on irrelevant areas. scSE is an attention mechanism block where the sSE block focuses on features in the spatial dimension, and the cSE block focuses on features in the channel dimension. The spatial attention maps and the channel attention maps are then generated, respectively. In previous studies, the scSE block was introduced to enhance the representation ability of the features by emphasizing the salient features of the targets and suppressing the background noise [39,40].

From the ablation experiment results, the multiple dilated convolution blocks and the attention mechanism blocks improved the accuracy effectively. The HDC block had the highest contribution to the accuracy improvement (Table 5), and we found that HDC was able to capture the details of building edges (Figure 13). However, we found that HDC misclassified some small targets in Figure 13c. Therefore, with the scSE block and the ASPP block added, we obtained more stable results which proved the advantages of the scSE block and the ASPP block in spatial context feature extraction and multi-scale feature fusion. Future research could explore deployable modules for rural land feature extraction in a more complex background.

### 4.4. Limitations of the Proposed Model

The ASCEND-UNet model achieved high accuracy in the rural settlement extraction task and successfully subdivided the rural settlements into DRSs and CRSs, but it also had limitations. First of all, in a complex background, such as those with shadows caused by vegetation and rivers, the edges of the buildings are blurred. It is difficult to accurately depict the edge of the building and uncertainty is introduced. To address this problem, high-resolution drone imagery offers distinctive advantages for the precise delineation of building boundaries in cluttered backgrounds with shadow effects. In our study, the edge detection accuracy was visually investigated based on segmentation results and satellite images. If the next step requires direct quantitative analysis of edge delineation accuracy, high-precision land survey data of rural settlements are needed, such as design drawings of single building. Secondly, due to the limitations in data acquisition, we took the Hangjiahu Plain as the study area. Tongxiang County is representative to a certain extent of the East China plain, but there are some different forms of rural settlements in the Shandong Province and Jiangsu Province. The extraction targets in our study are limited to two types of rural settlements. The application of multi-type rural settlement extraction in larger spatial areas remains a subject worth investigating. In the next step, we will try to further improve the UNet model and establish a broader dataset of rural land features. Finally, the ASCEND-UNet model integrates three modules, which makes the structure relatively complex. Our proposed ASCEND-UNet required a longer time for training and inference in the time complexity analysis, because it possessed more complex architecture with more parameters than UNet. Our priority goal was to classify two types of rural settlements accurately, and the model structure inevitably became complex. It is necessary to develop a model for rural land feature extraction that is easy to deploy and that can be applied in larger spatial regions.

## 5. Conclusions

Different types of rural settlements are formed during the implementation of the rural revitalization strategy, and they are mixed in their rural space. In this paper, a novel encoder–decoder network structure, ASCEND-UNet, was proposed for discriminating different types of rural settlements from remote sensing images. ASCEND-UNet was designed based on the original UNet with multiple dilated convolution blocks and attention mechanisms. This model had three key blocks; the ASPP block was used in the encoder, the scSE attention block was embedded at the skip connection, and the HDC block was utilized in the decoder. ASCEND-UNet was implemented to segment and distinguish dispersed and clustered rural settlements in VHR images of the Hangjiahu Plain, and achieved results of 96.05%, 95.15%, 95.59%, and 91.71% in precision, recall, F1-score, and MIoU, respectively. A series of model comparisons and ablation experiments verified the effectiveness of the proposed model. Compared with the original UNet model, ASCEND-UNet improved the accuracy of precision, recall, F1-score, and MIoU by 4.67%, 2.80%, 3.73%, and 6.28%, respectively. The HDC block was found to have the highest contribution to the accuracy improvement. This HDC model could obtain more accurate and stable results with the addition of scSE and ASPP blocks. ASCEND-UNet has enriched the automatic methods for semantic segmentation of different types of rural settlements in remote sensing images.

## Figures and Tables

**Table 1:** The difference between dispersed and clustered rural settlements.

Characteristic	Dispersed Rural Settlements (DRSs)	Clustered Rural Settlements (CRSs)
Composition	The main building, affiliated facilities (livestock pens and barns), gardens, woodlands, etc.	Neatly arranged standardized houses surrounded by less vegetation cover
Spatial morphology	Buildings are scattered and have various orientations, no distinct distribution pattern, low building density	Homogeneous building types, moderate or dense building density, orderly arrangement in space
Location	Close to country footpaths, rivers, and streams for transportation and water supply	Close to main roads for easy transportation

**Table 2:** The structural parameters of the ASCEND-Unet network.

Stage	Output Size	Template
Input	3 × 256 × 256	
Conv1	64 × 256 × 256	3×3 Conv,Stride=2
Maxpool	64 × 128 × 128	2×2 Maxpool,Stride=2
Conv2	128 × 128 × 128	(3×3 Conv+Relu)×2
Maxpool	128 × 64 × 64	2×2 Maxpool,Stride=2
Conv3	256 × 64 × 64	(3×3 Conv+Relu)×3
Maxpool	256 × 32 × 32	2×2 Maxpool,Stride=2
Conv4	512 × 32 × 32	(3×3 Conv+Relu)×3
Maxpool	512 × 16 × 16	2×2 Maxpool,Stride=2
Conv5	512 × 16 × 16	(3×3 Conv+Relu)×3
Up Layer1	512 × 16 × 16	2×2 Upsample $\left[\begin{array}{c} 3\times 3\;Conv,Stride=1,dilation_{rates}=1\\ 3\times 3\;Conv,Stride=1,dilation_{rates}=2\\ 3\times 3\;Conv,Stride=1,dilation_{rates}=5 \end{array}\right]\times 2$ BatchNormal
Up Layer2	512 × 32 × 32	2×2 Upsample $\left[\begin{array}{c} 3\times 3\;Conv,Stride=1,dilation_{rates}=1\\ 3\times 3\;Conv,Stride=1,dilation_{rates}=2\\ 3\times 3\;Conv,Stride=1,dilation_{rates}=5 \end{array}\right]\times 2$ BatchNormal
Up Layer3	256 × 64 × 64	2×2 Upsample $\left[\begin{array}{c} 3\times 3\;Conv,Stride=1,dilation_{rates}=1\\ 3\times 3\;Conv,Stride=1,dilation_{rates}=2\\ 3\times 3\;Conv,Stride=1,dilation_{rates}=5 \end{array}\right]\times 2$ BatchNormal
Up Layer4	128 × 128 × 128	2×2 Upsample $\left[\begin{array}{c} 3\times 3\;Conv,Stride=1,dilation_{rates}=1\\ 3\times 3\;Conv,Stride=1,dilation_{rates}=2\\ 3\times 3\;Conv,Stride=1,dilation_{rates}=5 \end{array}\right]\times 2$ BatchNormal
Up Layer5	64 × 256 × 256	2×2 Upsample $\left[\begin{array}{c} 3\times 3\;Conv,Stride=1,dilation_{rates}=1\\ 3\times 3\;Conv,Stride=1,dilation_{rates}=2\\ 3\times 3\;Conv,Stride=1,dilation_{rates}=5 \end{array}\right]\times 2$ BatchNormal
Segmentation Layer	3 × 256 × 256	1×1 Conv,Stride=1

**Table 3:** Accuracy assessment of different models.

Model	Precision (%)	Recall (%)	F1 (%)	MioU (%)
UNet	91.38	92.35	91.86	85.43
ASCEND-UNet	**96.05**	**95.15**	**95.59**	**91.71**
DeepLabv3+	87.90	86.02	86.94	78.06
PSPNet	83.02	74.99	78.63	67.32
SegNet	90.37	77.54	83.46	71.62

The best accuracy values are presented in bold.

**Table 4:** The computing time of the proposed ASCEND-UNet and other methods.

Model	Traning Time	Inference Time
UNet	~6.5 h	0 m 26 s
ASCEND-UNet	~7.5 h	0 m 30 s
Deeplabv3+	~4.6 h	0 m 25 s
PSPNet	~5.2 h	0 m 27 s
SegNet	~4.8 h	0 m 28 s

**Table 5:** Accuracy comparison of ablation experiments.

Model	Precision (%)	Recall (%)	F1-Score (%)	MioU (%)
UNet	91.38	92.35	91.86	85.43
UNet + scSE	95.40	94.54	94.97	90.61
UNet + ASPP	94.99	94.54	94.76	90.25
UNet + ASPP + HDC	95.88	94.87	95.27	91.32
UNet + scSE + ASPP	95.03	94.50	94.76	90.25
UNet + HDC	95.98	94.86	95.41	91.39
ASCEND-UNet	**96.05**	**95.15**	**95.59**	**91.71**

The best accuracy values are presented in bold.

## Data Availability

Data are contained within the article.
